# Serum Metabolomic Profiling of Piglets Infected with Virulent Classical Swine Fever Virus

**DOI:** 10.3389/fmicb.2017.00731

**Published:** 2017-04-27

**Authors:** Wenjie Gong, Junjie Jia, Bikai Zhang, Shijiang Mi, Li Zhang, Xiaoming Xie, Huancheng Guo, Jishu Shi, Changchun Tu

**Affiliations:** ^1^Department of Virology, Institute of Military Veterinary, Academy of Military Medical SciencesChangchun, China; ^2^Department of Anatomy and Physiology, College of Veterinary Medicine, Kansas State UniversityManhattan, KS, USA; ^3^Jiangsu Co-innovation Center for Prevention and Control of Important Animal Infectious Diseases and ZoonosesYangzhou, China

**Keywords:** metabolomic profiling, UPLC-MS, CSFV, metabolite

## Abstract

Classical swine fever (CSF) is a highly contagious swine infectious disease and causes significant economic losses for the pig industry worldwide. The objective of this study was to determine whether small molecule metabolites contribute to the pathogenesis of CSF. Birefly, serum metabolomics of CSFV Shimen strain-infected piglets were analyzed by ultraperformance liquid chromatography/electrospray ionization time-of-flight mass spectrometry (UPLC/ESI-Q-TOF/MS) in combination with multivariate statistical analysis. In CSFV-infected piglets at days 3 and 7 post-infection changes were found in metabolites associated with several key metabolic pathways, including tryptophan catabolism and the kynurenine pathway, phenylalanine metabolism, fatty acid and lipid metabolism, the tricarboxylic acid and urea cycles, branched-chain amino acid metabolism, and nucleotide metabolism. Several pathways involved in energy metabolism including fatty acid biosynthesis and β-oxidation, branched-chain amino acid metabolism, and the tricarboxylic acid cycle were significantly inhibited. Changes were also observed in several metabolites exclusively associated with gut microbiota. The metabolomic profiles indicate that CSFV-host gut microbiome interactions play a role in the development of CSF.

## Introduction

Classical swine fever (CSF) is a highly contagious swine infectious disease with high morbidity and mortality caused by CSFV, featuring high fever, severe depression, extensive hemorrhage, leucopenia, anorexia, and alternating constipation and diarrhea (Moennig et al., 2003). A notifiable disease of the OIE-World Organization for Animal Health, CSF causes major economic losses in many countries worldwide. CSFV replicates in organs with leucocytes, particularly mononuclear macrophages, as its target cells, causing malfunction or failure of multiple body components, including the immune, circulative, digestive, locomotor, reproductive, and respiratory systems (Floegel-Niesmann et al., 2003). While a detailed analysis of CSFV infection has identified various factors involved in viral replication and virulence (Ji et al., 2015), a comprehensive analysis of the host response to CSFV infection is necessary to decipher the underlying molecular mechanism of viral pathogenesis. Systems biology with high throughput techniques provides a powerful tool to reveal global changes in gene expression and metabolism associated with disease progression. Alterations in genomic expression profiles and the host proteome after CSFV infection have been determined using transcriptomics and proteomics in studies focused on elucidating alterations in upstream transcripts and proteins in virus-infected leukocytes, mononucleocytes, and PK-16 cells (Sun et al., 2008, 2010; Shi et al., 2009). However, global changes of small molecule metabolites in CSFV-infected piglets have not yet been reported.

Metabolomics is a powerful tool for capturing the global profiling of small molecule metabolites in a biological system and has been extensively used to assess the physiological and pathological status of human and animals (Lu et al., 2012; Guo et al., 2015). Mounting evidence indicates that the metabolome obtained under specific conditions displays unique chemical characterizations, thereby showing great promise for biomarker identification and diagnosis of diseases such as cancers and neurodegenerative and viral diseases (Cui et al., 2013; Sun et al., 2013; Ni et al., 2014). Indeed, small molecule metabolites are the end products of cellular processes and can directly influence the phenotype to a greater extent than transcripts and proteins (Zhou et al., 2012; Cui et al., 2013; Sun et al., 2013). So far, LC-MS or GC-MS-based metabolomics have been used to capture global changes in small molecule metabolites in the body fluids of patients infected with HIV, HCV, HBV, and dengue virus with some of the significantly altered metabolites serving as potential biomarkers of these viral diseases (Sun et al., 2013; Cui et al., 2013; Zhou et al., 2012). By identifying perturbed biochemical networks and metabolic pathways, metabolomic studies might also provide targets for future disease therapy.

In the present study, serum samples from highly virulent CSFV-infected piglets and healthy control were subjected to metabolomics analysis using ultraperformance liquid-chromatography/electrospray ionization-time of flight-mass spectrometry (UPLC/ESI-Q-TOF/MS). We show that the serum metabolome obtained through multivariate statistical analysis can separate CSFV-infected piglets from healthy control animals, and that the altered metabolic pathways may be associated with disease development and progression of CSF.

## Experimental section

### Animal experiments

Weaning piglets of (30 days old; *n* = 20), free of porcine reproductive and respiratory syndrome virus (PRRSV), bovine viral diarrhea virus (BVDV), porcine circovirus type 2 (PCV-2), pseudorabies virus (PRV), and porcine parvovirus (PPV), were purchased from Jiangshu Yongkang Husbandry Company (Jiangshu, China). The piglets were randomly divided into two groups of 10 (infected and control) and housed in a room of negative-pressure facilities in Sinovet Company (Jiangshu, China), five piglets per pen. The animals were allowed free access to standard diet and water. The animal studies were approved by the Experimental Animal Use and Care Committee, Academy of Military Medical Sciences, China.

After a 7-day observation period piglets in the infected group were intramuscularly injected with 10^6^ TCID_50_ of the highly virulent Shimen strain of CSFV with controls receiving an equal volume of PBS. Rectal temperatures were recorded each morning and animals were observed daily for clinical signs. Leukocyte counts were measured on whole blood samples collected from piglets at 0, 3, and 7 days post-infection (dpi) using a Medonic CA 620 counter (Boule Medical AB, Stockholm, Sweden). Viremia of whole blood collected from CSFV-infected piglets at dpi 3 and 7 were measured by real-time PCR as described previously (Shi et al., 2009).

### Serum collection

Blood was collected via the precaval vein into BD Vacutainer Rapid Serum Tubes (BD, Franklin Lakes, New Jersey, USA) and serum was harvested by centrifugation at 2,000 g for 10 min at 4°C. Samples were stored at −80°C until use.

### UPLC/ESI-Q-TOF/MS analysis of serum extracts

For UPLC/ESI–Q-TOF/MS analysis, a 200 μl aliquot of serum was mixed with 600 μl methanol, vortexed for 1 min, and centrifuged at 12,000 g for 15 min at 4°C. A 5 μl aliquot of the resulting supernatant was injected into a 100 × 2.1 mm^2^, 1.8 μm HSS T3 column (Waters, Milford, MA) held at 40°C using an Agilent 1290 Infinity UPLC system (Santa Clara, CA). The binary gradient elution system consisted of (A) water (containing 0.1% formic acid, v/v) and (B) acetonitrile (containing 0.1% formic acid, v/v) and separation was achieved using a linear gradient which was run from 5% to 95% B over 30 min at 0.35 ml/min. All samples were kept at 4°C during the analysis. The mass spectrometric data was collected using an Agilent 6538 UHD and Accurate-Mass Q-TOF/MS system equipped with an electrospray ionization (ESI) source operating in either positive or negative ion mode. Two reference masses were continuously infused to the system to allow constant mass correction during the run, with reference masses in the positive and negative ion modes of m/z 121.0509 and 922.0098, and m/z 112.985587 and 1033.988109 respectively. The ESI source parameters were: capillary voltage of 4.0 kV for positive mode and 3.5 kV for negative mode, gas temperature of 350°C, gas flow of 11 L/min, nebulizer of 45 psi, fragmentor of 120 V, skimmer of 60 V, and octopole RF Peak of 750 V.

### Data processing and analysis

The raw ULPC-MS ESI data were converted to mzXML using MassHunter Workstation software from Agilent (Santa Clara, CA, USA), and the file was then imported into XCMS software in the R platform (Scripps, La Jolla, CA, USA) for preprocessing, including peak picking, alignment, integration and retention time (RT) alignment. An Excel file was obtained with three dimension data sets including m/z, peak RT, and normalized peak intensities, and RT–m/z pairs were used as the identifier for each ion. The multivariate data matrix was analyzed with Simca-P 11.0 (Umetrics, Umea, Sweden). To reveal metabolic changes occurring following infection, the unsupervised method (primary component analysis, PCA) was utilized to provide an overview of systemic variations and general clustering among all groups, and the supervised method (partial least-square discriminant analysis, PLS-DA) was utilized to identify differential metabolites accounting for the separation between groups of piglets. Potential differential metabolites were selected according to the Variable Importance in the Projection (VIP) values and the variables with VIP>1 were considered to be influential for the separation of samples in PLS-DA analysis. In addition, the Student's *t*-test was applied to determine if the differential metabolites obtained from PLS-DA modeling were statistically significant (*p* < 0.05) among groups at the univariate analysis level.

Identification of differential metabolites was performed as following: first, the element composition of the specific m/z ion was calculated based on the exact mass, the nitrogen rule and the isotype pattern by Masshunter software from Agilent. Then, the elemental composition and exact mass were used for open source database searching, including METLIN (http://metlin.scripps.edu/). In addition, Kyoto Encyclopedia of Genes and Genomes (http://www.genome.jp/kegg/) was used to identify the altered metabolic pathways in piglets following CSFV infection.

## Results

### Clinical presentations of piglets infected with CSFV

**Figure 1:**
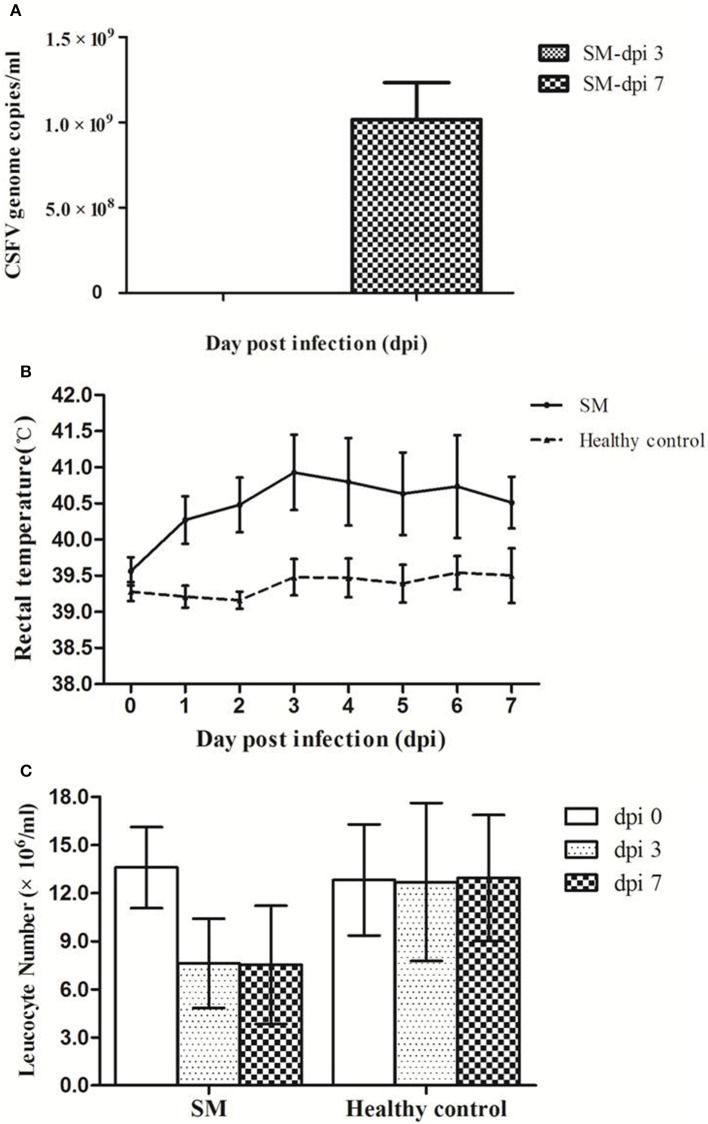
**Pigs infected with CSFV Shimen strain developed viremia, high fever, and leucopenia**. Pigs were infected with CSFV Shimen strain (SM) and healthy control pigs were treated with PBS. Viremia was detected only in Shimen-infected piglets at dpi 3 and dpi 7 **(A)**. High fever (>40.5°C, **B**) and leucopenia **(C)** occurred in piglets infected with CSFV only.

After infection with CSFV virulent Shimen strain, viremia was detected at low level (10^5.0^ genome copies/ml) in piglets at dpi 3 and reached high level at about 10^9.0^ genome copies/ml at dpi 7 (Figure 1A). Typical CSF characteristics including high fever and leukopenia were observed in CSFV-infected piglets. As shown in Figure 1B, the rectal temperature of infected piglets (SM) increased from 39.6°C at dpi 0 to ≥40.3°C at dpi 1, peaking at dpi 3 (40.9°C), while no significant changes in rectal temperatures were observed in the controls. In addition, leukocyte counts in infected piglets dropped by ~50% by dpi 3 and dpi 7 while control levels remained unchanged (Figure 1C). Alternating constipation and diarrhea, convulsions, depression, and coma were also observed in CSFV-infected piglets. Of note is that one of the infected piglets did not show any clinical signs until dpi 8, while serum samples collected at dpi 3 and 7 from this animal were included in the metabolomic analysis.

### Metabolomic analysis of serum metabolites

**Figure 2:**
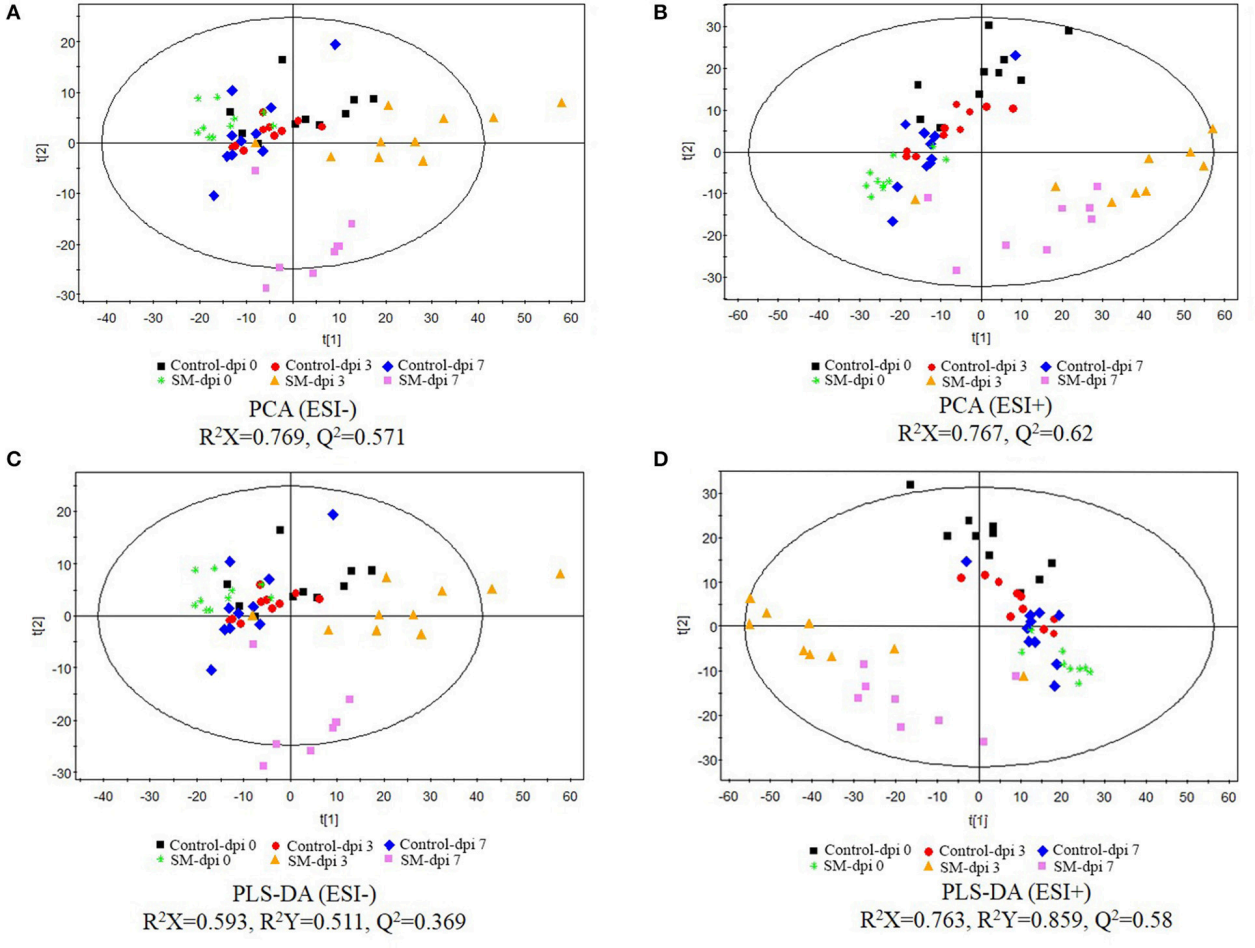
**Score plots of principal component analysis (PCA) and partial least-square discriminant analysis (PLS-DA) of serum extracts from healthy controls and piglets infected with CSFV**. The principal component analysis (PCA) (**A**, negative ion mode-ESI-; **B**, positive ion mode-ESI+) and partial least-square discriminant analysis (PLS-DA) (**C**, negative ion mode-ESI-; **D**, positive ion mode-ESI+) models were constructed using UPLC-MS metabolomics data from healthy subjects and CSFV-infected piglets (SM) at three different time points. Results indicate the separation of healthy subjects and infected piglets. The ellipses represent 95% confidence intervals of all samples.

Serum metabolomic profiles following CSFV infection were characterized using UPLC/ESI-Q-TOF/MS, and PCA and PLS-DA were applied to the data. QC data showing no drift of stable retention time in all peaks indicated that the quality of data was acceptable for the following metabolomic analysis. PCA score plots revealed clear separation between the uninfected and infected piglets under both positive and negative modes (Figures 2A,B). Further, distinct metabolic profiles in infected animals were observed before (dpi 0) and after infection (dpi 3 and 7), but no significant separation was observed in the control.

The UPLC-MS data were further processed by a PLS-DA scores plot. The quality of the PLS-DA model was evaluated by *R*^2^*Y* and *Q*^2^ parameters, which indicated the fitness and prediction ability, respectively. PLS-DA score plots revealed excellent separation of the uninfected and infected piglets (Figures 2C,D). The first 2-component PLS-DA scores plot of serum metabolites showed that infected piglets were clearly distinguished from uninfected ones by the principal component *t*[1].

### Effect of infection duration on metabolite profile in CSFV-infected piglets

**Figure 3:**
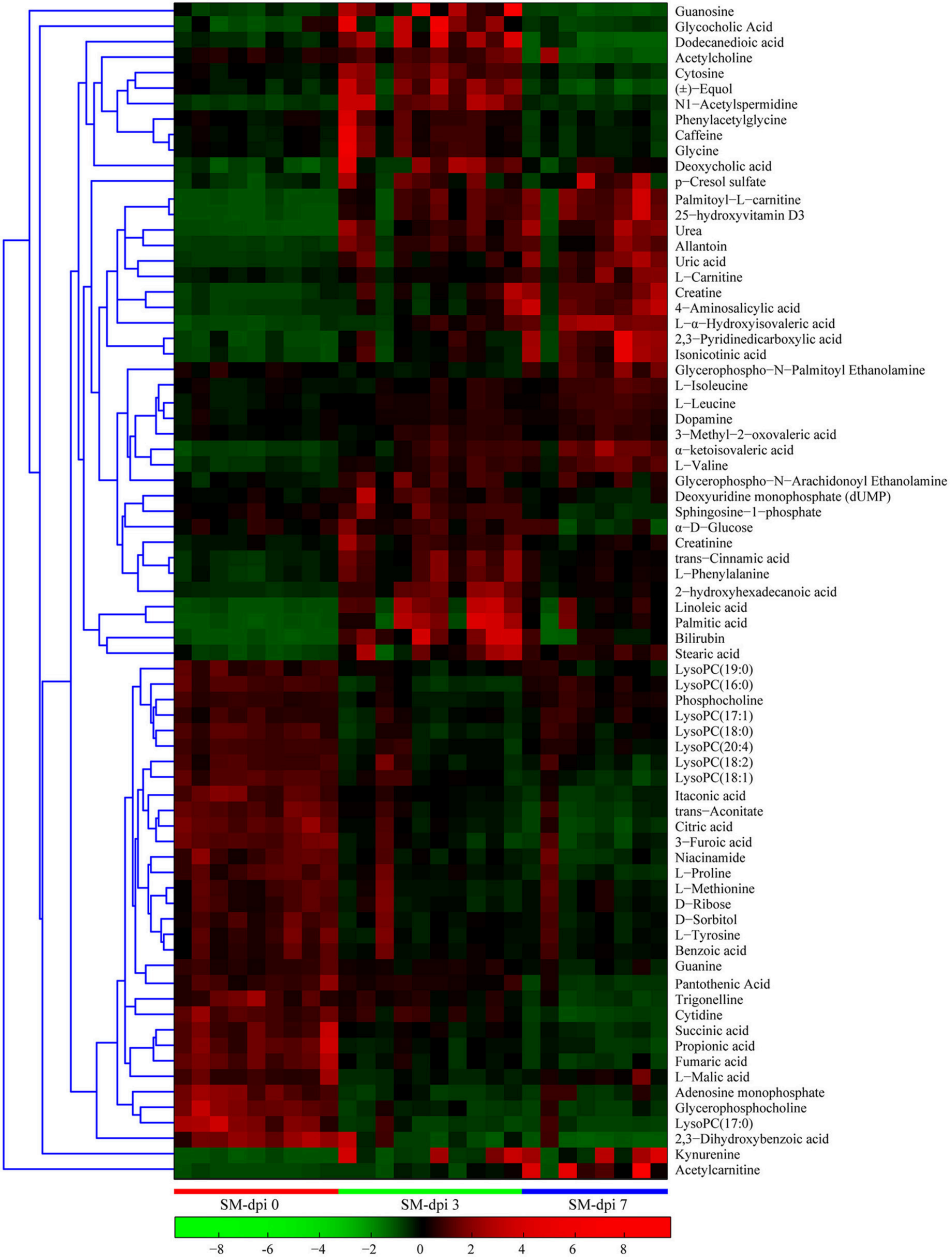
**Heat map of differentially expressed metabolites in piglets infected with CSFV**. Each row shows ion intensity plots of specific metabolites in the serum collected from healthy subjects and CSFV challenged piglets (SM). The dendrogram in the left side of the heat map was generated by using MATLAB 7.5. The leaf nodes of this dendrogram represent individual metabolites and the remaining nodes represent the clusters to which the metabolites belong. The distance between merged clusters is monotone increasing with the level of the merger. The horizontal distance of each node in the plot is proportional to the value of the intergroup dissimilarity between its two daughter nodes.

Differential metabolites obtained through UPLC/ESI-Q-TOF/MS were selected based on the criteria VIP > 1 in OPLS-DA analysis and *P* < 0.05 in the Student's *t*-test (Table 1). The changed metabolites were associated with several key metabolic pathways, including tryptophan catabolism and the kynurenine pathway, phenylalanine metabolism, fatty acid and lipid metabolism, the citric acid and urea cycles, and branched-chain amino acid, and nucleotide metabolism. Different stages of CSFV infection had a significant impact on the metabolite profiles, as reflected in both the number and fold changes of altered serum metabolites detected at dpi 3 and 7 (Table 1). These differential metabolites show three different time-course trends: elevated-elevated, elevated-decreased, and decreased-decreased, as depicted in the hierarchical clustering heat map (Figure 3).

**Table 1:** **Differential metabolites in CSFV-infected piglets compared to healthy controls**.

**Metabolites**	**Pathway**	**VIP[1]**	***t*-test**	**FC (SM-dpi 3 vs. SM-dpi 0)**	**VIP[1]**	***t*-test**	**FC (SM-dpi 7 vs. SM-dpi 0)**	**Mode**
Bilirubin	Heme catabolism	1.26	1.70E-05	17.24	1.10	1.90E-03	6.22	ESI+
L-Ornithine	Arginine and proline metabolism				1.26	1.53E-04	−1.96	ESI+
Citrulline	Arginine and proline metabolism				1.38	1.76E-05	−1.78	ESI−
Creatine	Arginine and proline metabolism	0.95	4.16E-03	3.21	1.37	9.73E-06	5.06	ESI+
Creatinine	Arginine and proline metabolism	1.10	5.23E-04	1.99	0.92	1.41E-02	1.29	ESI+
Urea	Arginine and proline metabolism	1.27	1.05E-05	8.96	1.34	2.32E-05	10.70	ESI+
L-Proline	Arginine and proline metabolism	1.14	2.59E-04	−1.68	1.34	2.49E-05	−2.35	ESI+
Uric acid	Purine metabolism	1.16	5.59E-04	2.51	1.36	2.64E-05	4.22	ESI−
Allantoin	Purine metabolism	1.42	9.97E-07	3.84	1.32	7.40E-05	3.94	ESI−
p-Cresol sulfate	Aromatic compounds	1.09	1.54E-03	3.15	0.95	1.25E-02	3.37	ESI−
L-Phenylalanine	Phenylalanine metabolism	1.27	1.16E-05	2.81	1.34	2.55E-05	1.80	ESI+
Phenylacetylglycine	Phenylalanine metabolism	0.78	3.53E-02	1.67	0.95	1.31E-02	−1.45	ESI−
Benzoic acid	Phenylalanine metabolism	0.84	1.41E-02	−1.48	1.02	5.01E-03	−1.52	ESI+
Glycine	Glycine, serine and threonine metabolism	0.75	4.31E-02	1.64	0.98	9.39E-03	−1.50	ESI−
DL-2-Aminooctanoic acid	Amino compound				1.22	3.43E-04	−4.48	ESI+
4-Hydroxybenzenesulfonic acid	Aromatic amino acid				0.86	2.64E-02	15.15	ESI−
Hippuric acid	Phenylalanine metabolism				0.88	2.04E-02	−2.00	ESI+
L-Tyrosine	Phenylalanine metabolism	0.84	1.40E-02	−1.63	1.02	5.35E-03	−1.66	ESI−
Dopamine	Phenylalanine metabolism	1.10	4.71E-04	1.34	1.29	8.59E-05	1.55	ESI+
trans-Cinnamic acid	Phenylalanine metabolism	1.27	1.23E-05	2.85	1.34	2.24E-05	1.75	ESI+
4-Aminosalicylic acid	Phenylalanine metabolism	0.83	1.51E-02	2.36	1.31	5.02E-05	5.36	ESI+
2,3-Dihydroxybenzoic acid	Benzoic acid metabolism	1.18	3.78E-03	−2.87	1.50	2.26E-07	−9.40	ESI−
D-Ribose	Pentose phosphate pathway	0.89	8.73E-03	−1.63	1.00	6.78E-03	−1.53	ESI+
Gluconic acid	Pentose phosphate pathway				1.13	1.81E-03	−1.52	ESI−
α-D-Glucose	Glycolysis/Gluconeogenesis	0.76	2.90E-02	1.31	0.95	1.11E-02	−2.14	ESI+
L-Lactic acid	Glycolysis/Gluconeogenesis				1.25	2.98E-04	−2.25	ESI−
D-Sorbitol	Glucose metabolism	1.04	2.77E-03	−1.60	1.23	4.03E-04	−1.77	ESI−
Itaconic acid	TCA cycle	1.42	9.92E-07	−2.07	1.48	5.81E-07	−3.19	ESI−
Citric acid	TCA cycle	1.44	4.77E-07	−2.16	1.54	2.79E-08	−4.06	ESI−
trans-Aconitate	TCA cycle	1.45	4.03E-07	−1.93	1.47	9.38E-07	−3.06	ESI−
Succinic acid	TCA cycle	1.19	3.57E-04	−1.99	1.34	4.62E-05	−3.36	ESI−
L-Malic acid	TCA cycle	1.33	1.70E-05	−2.40	1.38	1.65E-05	−3.01	ESI−
Fumaric acid	TCA cycle	1.32	2.16E-05	−2.26	1.42	5.53E-06	−3.53	ESI−
Oxaloacetate	TCA cycle				1.02	4.95E-03	2.35	ESI+
Pantothenic Acid	Beta-Alanine metabolism	0.89	1.47E-02	−1.28	1.40	1.09E-05	−2.91	ESI−
L-Ascorbic acid	Glutathione metabolism				1.14	1.58E-03	−6.72	ESI−
Adenosine monophosphate	Purine metabolism	1.44	5.85E-09	−3.71	1.31	4.64E-05	−2.39	ESI+
1-Methyladenosine	Methylation				1.40	3.98E-06	−1.80	ESI+
Cytidine	Pyrimidine metabolism	1.06	1.00E-03	−1.53	1.40	4.04E-06	−1.99	ESI+
Cytosine	Pyrimidine metabolism	1.16	1.52E-04	2.12	1.20	4.18E-04	−1.57	ESI+
3-Methylcytosine	Pyrimidine metabolism				1.22	2.97E-04	−1.65	ESI+
Deoxyuridine monophosphate (dUMP)	Pyrimidine metabolism	0.85	1.29E-02	1.62	1.15	1.03E-03	−1.91	ESI+
Guanine	Purine metabolism	1.04	1.26E-03	−1.28	1.30	5.81E-05	−1.71	ESI+
Guanosine	Purine metabolism	0.95	4.45E-03	5.33	1.04	4.00E-03	−2.93	ESI+
Inosine	Purine metabolism	0.84	1.44E-02	4.75				ESI+
Caffeine	Caffeine metabolism	0.76	2.97E-02	2.12	0.89	1.81E-02	−1.37	ESI+
Kynurenine	Tryptophan metabolism	0.86	1.11E-02	10.13	1.08	2.61E-03	12.63	ESI+
Quinolinic acid	Tryptophan metabolism	1.10	1.41E-03	2.53	1.24	3.50E-04	6.08	ESI−
Serotonin	Tryptophan metabolism				1.38	7.23E-06	−6.90	ESI+
Isonicotinic acid	Tryptophan metabolism	1.07	2.07E-03	2.38	1.22	5.06E-04	5.43	ESI−
3-Furoic acid	Tryptophan metabolism	1.42	9.79E-07	−2.26	1.45	2.04E-06	−2.97	ESI−
Trigonelline	Tryptophan metabolism	1.04	1.30E-03	−1.54	1.40	4.29E-06	−3.66	ESI+
Niacinamide	Tryptophan metabolism	1.16	1.49E-04	−1.93	1.13	1.26E-03	−2.15	ESI+
Indoleacetaldehyde	Tryptophan metabolism				1.38	6.76E-06	−6.74	ESI+
3-Indolepropionic acid	Tryptophan metabolism				1.22	3.27E-04	−5.91	ESI+
Indoxylsulfuric acid	Tryptophan metabolism				1.37	2.48E-05	2.91	ESI−
L-Isoleucine	Branched chain amino acids (BCAA)	0.74	3.31E-02	1.23	1.27	1.18E-04	1.69	ESI+
L-Leucine	Branched chain amino acids	1.15	2.00E-04	1.42	1.41	3.32E-06	1.75	ESI+
L-Valine	Branched chain amino acids	1.39	1.47E-07	2.52	1.43	1.42E-06	3.11	ESI+
3-Methyl-2-oxovaleric acid	BCAA metabolism	1.06	2.39E-03	1.37	1.47	9.33E-07	1.97	ESI−
α-ketoisovaleric acid	BCAA metabolism	1.40	2.48E-06	3.39	1.47	1.03E-06	4.88	ESI−
L-α-Hydroxyisovaleric acid	BCAA metabolism	1.27	8.16E-05	4.15	1.45	1.53E-06	8.41	ESI−
(±)-Equol	Aromatic compounds	1.22	1.83E-04	2.84	1.31	9.51E-05	−3.02	ESI−
Acetylcarnitine	Carnitine	0.98	2.85E-03	2.28	0.97	9.02E-03	10.75	ESI+
L-Carnitine	Carnitine	1.11	3.90E-04	1.50	1.24	2.23E-04	2.38	ESI+
Butyryl-L-carnitine	Carnitine				1.30	6.43E-05	5.63	ESI+
Palmitoyl-L-carnitine	Carnitine	1.31	2.99E-06	7.36	1.33	3.22E-05	10.74	ESI+
Propionyl-L-carnitine	Carnitine	1.10	4.96E-04	1.80				ESI+
Oleamide	Acyl amide	1.13	3.00E-04	1.59	1.02	5.45E-03	1.19	ESI+
Oleic Acid	Unsaturated fatty acids	1.07	7.56E-04	6.26				ESI+
Linoleic acid	Unsaturated fatty acids	1.30	3.59E-05	10.78	1.31	8.39E-05	5.91	ESI−
Arachidonic Acid (peroxide free)	Unsaturated fatty acids	1.05	2.51E-03	1.58				ESI−
Eicosapentaenoic Acid	Unsaturated fatty acids				1.07	2.85E-03	−1.55	ESI+
Docosahexaenoic Acid ethyl ester	Unsaturated fatty acids	0.89	8.45E-03	8.63				ESI+
LTB5	Unsaturated fatty acids	1.15	1.84E-04	10.28				ESI+
Palmitic acid	Fatty acids	1.20	2.66E-04	17.79	1.27	2.18E-04	8.25	ESI−
Stearic acid	Fatty acids	1.11	1.16E-03	4.74	1.54	2.59E-08	3.95	ESI−
Propionic acid	Short chain fatty acids	1.30	4.24E-05	−2.48	1.38	1.67E-05	−4.00	ESI−
2-hydroxyhexadecanoic acid	Hydroxy fatty acids	1.40	2.66E-06	3.24	1.45	1.55E-06	1.84	ESI−
2-Linoleoyl Glycerol	Acyl glycerol	1.19	8.13E-05	1.61	1.22	3.28E-04	1.33	ESI+
Arachidonoyl dopamine	TRPV1 ligand				1.14	1.56E-03	3.12	ESI−
Taurine	Taurine				1.20	6.27E-04	−1.64	ESI−
Glycerophospho-N-Arachidonoyl Ethanolamine	Acyl amide	1.08	6.47E-04	2.07				ESI+
L-Methionine	Methionine metabolism	0.95	4.25E-03	−1.73	0.98	8.27E-03	−1.55	ESI+
N1-Acetylspermidine	Methionine metabolism	1.27	1.01E-05	5.46	0.86	2.46E-02	1.27	ESI+
Histamine	Histidine metabolism				1.34	2.59E-05	−8.15	ESI+
(R)-(+)-2-Pyrrolidone-5-carboxylic acid	Derivative of glutamic acid				0.78	4.94E-02	1.59	ESI−
N-Acetyl-L-glutamic acid	Derivative of glutamic acid	0.91	7.06E-03	1.53				ESI+
L-Threonine	Glycine, serine and threonine metabolism				1.16	1.13E-03	−2.16	ESI−
Sphingosine	Sphingolipid metabolism				1.13	1.35E-03	−5.62	ESI+
Sphinganine	Sphingolipid metabolism				1.12	1.42E-03	−5.14	ESI+
Sphingosine-1-phosphate	Sphingolipid metabolism	0.98	2.89E-03	1.46	1.36	1.30E-05	−1.80	ESI+
Glycerophosphocholine	Choline metabolism	1.41	3.44E-08	−3.57	1.46	4.47E-07	−3.96	ESI+
Phosphocholine	Choline metabolism	1.38	1.62E-07	−1.80				ESI+
Acetylcholine	Choline metabolism	1.06	9.49E-04	1.44	1.28	9.83E-05	−5.49	ESI+
cholesterol sulfate	Steroid hormone biosynthesis	0.87	1.03E-02	4.34				ESI+
desoxycorticosterone acetate	Steroid hormone biosynthesis	1.03	3.41E-03	1.63				ESI−
Cortisol	Steroid hormone biosynthesis				1.15	9.58E-04	2.11	ESI+
25-hydroxyvitamin D3	Steroid hormone biosynthesis	1.33	1.49E-06	8.00	1.34	2.43E-05	11.66	ESI+
Glycocholic Acid	Bile acids	0.85	1.22E-02	4.24	1.23	2.49E-04	−1.61	ESI+
Glycodeoxycholate	Bile acids	0.86	1.83E-02	3.22				ESI−
Deoxycholic acid	Bile acids	1.11	1.22E-03	6.26	1.16	1.18E-03	3.18	ESI−
Dodecanedioic acid	Dicarboxylic acid	1.19	3.45E-04	3.97	1.23	4.23E-04	−3.87	ESI−
LysoPC(14:0)	Glycerophospholipid metabolism				1.47	2.37E-07	−2.11	ESI+
LysoPC(15:0)	Glycerophospholipid metabolism				1.42	1.65E-06	−3.26	ESI+
LysoPC(16:0)	Glycerophospholipid metabolism	1.44	3.99E-09	−2.77	1.20	4.21E-04	−1.34	ESI+
LysoPC(17:0)	Glycerophospholipid metabolism	1.34	1.08E-06	−4.49	1.38	7.41E-06	−4.74	ESI+
LysoPC(17:1)	Glycerophospholipid metabolism				1.46	3.68E-07	−2.76	ESI+
LysoPC(18:0)	Glycerophospholipid metabolism	1.36	3.83E-07	−2.32	1.19	5.70E-04	−1.50	ESI+
LysoPC(18:1)	Glycerophospholipid metabolism	1.24	2.55E-05	−1.69	1.30	5.91E-05	−1.83	ESI+
LysoPC(18:2)	Glycerophospholipid metabolism	1.03	1.54E-03	−1.65	1.31	5.44E-05	−1.87	ESI+
LysoPC(19:0)	Glycerophospholipid metabolism	1.33	1.80E-05	−1.69	1.24	3.63E-04	−1.69	ESI+
LysoPC(20:1)	Glycerophospholipid metabolism	1.45	2.28E-09	−2.28				ESI+
LysoPC(20:2)	Glycerophospholipid metabolism	1.24	2.87E-05	−1.56				ESI+
LysoPC(20:4)	Glycerophospholipid metabolism	1.28	8.12E-06	−1.80	1.42	2.30E-06	−1.59	ESI+

*VIP, Variable Importance in the Projection; FC, fold change; SM, piglets infected with CSFV Shimen strain; dpi, day post infection*.

**Figure 4:**
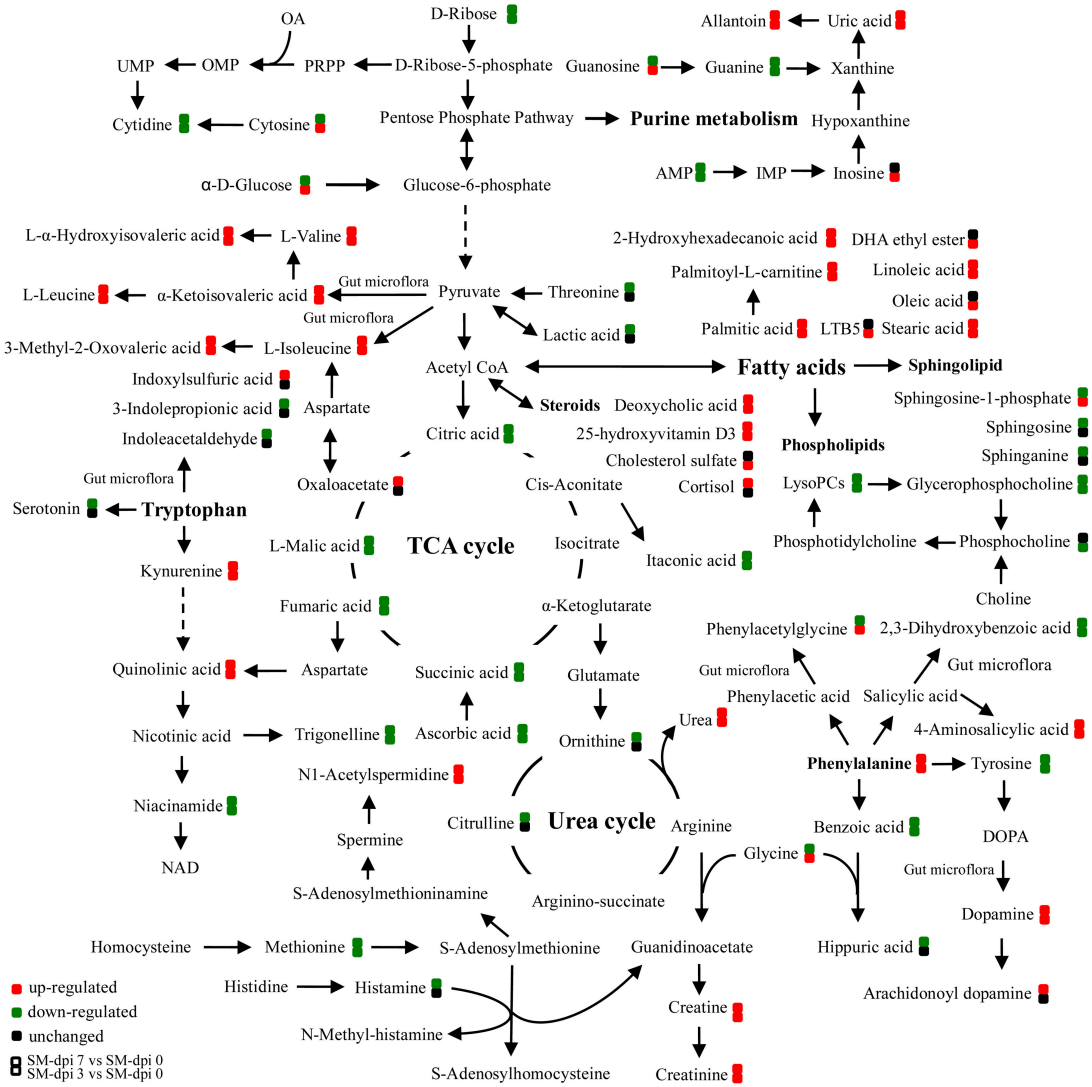
**Schematic representation of altered metabolic pathways in piglets infected with CSFV**. Serum metabolomics of CSFV-infected piglets (SM) were performed by UPLC-MS and many metabolites and associated metabolic pathways were significantly changed with the development of CSF, including tryptophan/kynurenine metabolism, fatty acid biosynthesis, TCA and urea cycles, amino acids, and nucleotide metabolism. Red: up-regulation; green: down-regulation.

Specifically, a total of six saturated and unsaturated long chain fatty acids, 2 acylcarnitines, 3 branched-chain amino acids (BCAAs) and their derivatives (L-α-hydroxyisovaleric acid, α-ketoisovaleric acid, and 3-methyl-2-oxovaleric acid), phenylalanine and its derivatives (trans-cinnamic acid, dopamine, and 4-aminosalicylic acid), tryptophan derivatives (kynurenine, quinolinic acid, and isonicotinic acid), urea, uric acid, allantoin, *p*-cresol sulfate, creatine, creatinine, and bilirubin showed an elevated trend at both dpi 3 and 7 (Figure 4 and Table 1). Three steroids (cortisol, 25-hydroxyvitamin D3, cholesterol sulfate) also showed an upward trend during CSFV infection (Figure 4 and Table 1). Conversely, phospholipids, 5 intermediates in the TCA cycle and 2 intermediates in the urea cycle, 2 nucleosides (cytidine and guanine), 3 quinolinic acid derivatives (trigonelline, niacinamide, and 3-furoic acid), amino acids (threonine, tyrosine, and methionine), and 2,3-dihydroxybenzoic acid displayed a downward trend (Figure 4 and Table 1). Guanosine, cytosine, glycocholic acid, deoxycholic acid, acetylcholine, equol, N1-acetylspermidine, caffeine, glycine, phenylacetylglycine, sphingosine-1-phosphate, and α-D-glucose showed an increasing trend at dpi 3 but normalized to control levels by dpi 7 (Figure 4 and Table 1).

### Altered energy and lipid metabolism in CSFV-infected piglets

The TCA cycle is important in energy production for the biochemical processes in the body. Intermediates, including citric, succinic, fumaric, and malic acids, decreased as the infection developed except for an up-regulation of oxaloacetate in the late stages (Figure 4), indicating the blocking of energy production in CSFV infected piglets. Substantial levels of saturated fatty acids (palmitic, 2-hydroxyhexahecanoic and stearic acids), and unsaturated fatty acids (oleic, LTB5, and linoleic acids), as well as acyl carnitines, including palmitoyl-L-carnitine, acetylcarnitine and butyryl-L-carnitine accumulated in the serum of infected piglets (Figure 4 and Table 1).

Accumulation of these compounds may indicate that energy production through fatty acid β-oxidation was perturbed. Another energy production pathway, degradation of branched-chain amino acids (BCAA) to acetyl-CoA and succinyl CoA, may also be inhibited because of the accumulation of BCAAs (valine, leucine, and isoleucine) and their derivatives after CSFV infection. Other amino acids associated with energy production, such as methionine and threonine, showed a downward trend in infected piglets (Figure 4 and Table 1). Furthermore, a short chain fatty acid, propionic acid, which is also used for energy production by gut bacteria, was down-regulated during infection (Table 1). In addition, pantothenic acid used for the synthesis of coenzyme A was found to be first increased at dpi 3, then decreased at dpi 7 (Table 1). Taken together, energy metabolism was significantly inhibited in piglets infected with the highly virulent CSFV strain.

### Altered aromatic amino acid metabolism in CSFV–infected piglets

Indole-containing compounds 3-indolepropionic acid (IPA) and indoleacetaldehyde were down-regulated at dpi 7 in infected piglets, but another indole derivative, indoxylsulfuric acid, was elevated (Table 1). Production of these indole derivatives is associated with gut microbiotic metabolism. In particular, IPA is a metabolite produced by certain types of intestinal bacteria, and its decrease may reflect perturbation of gut microflora by CSFV infection.

The metabolism of another indole-containing compound tryptophan was also significantly altered in CSFV-infected piglets. Tryptophan is an essential amino acid for animals and can be metabolized through several pathways. One key route, the tryptophan-kynurenine pathway, was activated following CSFV infection, as reflected in the increased serum levels of kynurenine and its derivative quinolinic acid, concomitantly with down-regulation of quinolinic acid derivatives (trigonelline and niacinamide) (Figure 4 and Table 1). Likewise, a metabolite in another tryptophan metabolism pathway, serotonin, was significantly decreased during infection (Table 1).

Phenylalanine metabolism was also changed by infection. Phenylalanine derivatives, including phenylacetylglycine and hippuric acid were down-regulated, but metabolite 4-aminosalicylic acid produced from phenylalanine degradation was significantly elevated during infection (Figure 4 and Table 1), an observation consistent with the increased levels of phenylalanine. Further, perturbation of phenylalanine metabolism to tyrosine was observed during CSFV infection, but the tyrosine derivative, dopamine, was unexpectedly increased.

Equol, the gut bacteria-regulated metabolite from daidzein, was observed to be elevated at dpi 3 and decreased at dpi 7 following infection (Table 1), a change that may further reflect the perturbation of the gut microbiota induced by CSFV infection. Altogether, the above results clearly implicate the involvement of gut microorganims in the serum metabolite profiles in CSFV-infected piglets.

### Altered nucleotide metabolism and urea cycle in CSFV-infected piglets

Nucleotides cytidine and guanine were down-regulated during CSFV infection as well as AMP, but cytosine and guanosine were found to be elevated at dpi 3 and subsequently down-regulated at dpi 7. Although inosine was found to be elevated only in the early stages of CSFV infection, its derivatives uric acid and allantoin were significantly up-regulated throughout.

In addition to the alterations in nucleotide metabolism, nitrogen homeostasis was also perturbed during infection, as evidenced by the decreased levels of citrulline and ornithine and the elevation of urea. Further, metabolism of arginine to creatine and creatinine was elevated. This is an important observation since urea and creatinine accumulated in the serum are indicators of kidney failure. Also, the elevated levels of uric acid, allantoin and the gut bacteria-regulated metabolite *p*-cresol sulfate reflect the occurrence of uremia in CSFV-infected piglets.

## Discussion

In the present study, we analyzed the serum metabolomic profiles of CSFV-infected piglets using UPLC/ESI–Q-TOF-MS. PLS-DA modeling and clustering revealed significant differences in the global serum metabolite profiles between healthy controls and animals infected with the highly virulent CSFV Shimen strain. Metabolites in energy metabolism and aromatic amino acid metabolism were especially affected following infection. Further, serum metabolite profiles of CSFV-infected piglets clearly demonstrate that the perturbation of host metabolites or metabolic pathways is associated with microbial metabolism, especially the composition and function of gut microbiota. In previous proteomics studies regarding CSFV Shimen infection, only metabolic enzymes involved in glycolysis and TCA cycle, including glyceraldehyde 3-phosphate dehydrogenase (GAPDH), phosphoglycerate mutase (PGAM1), and malate dehydrogenase, were found to be differentially expressed among altered proteins (Sun et al., 2008, 2010). Therefore, this study provided more information about alteration of metabolites and associated pathways in piglets following CSFV infection.

**Figure 5:**
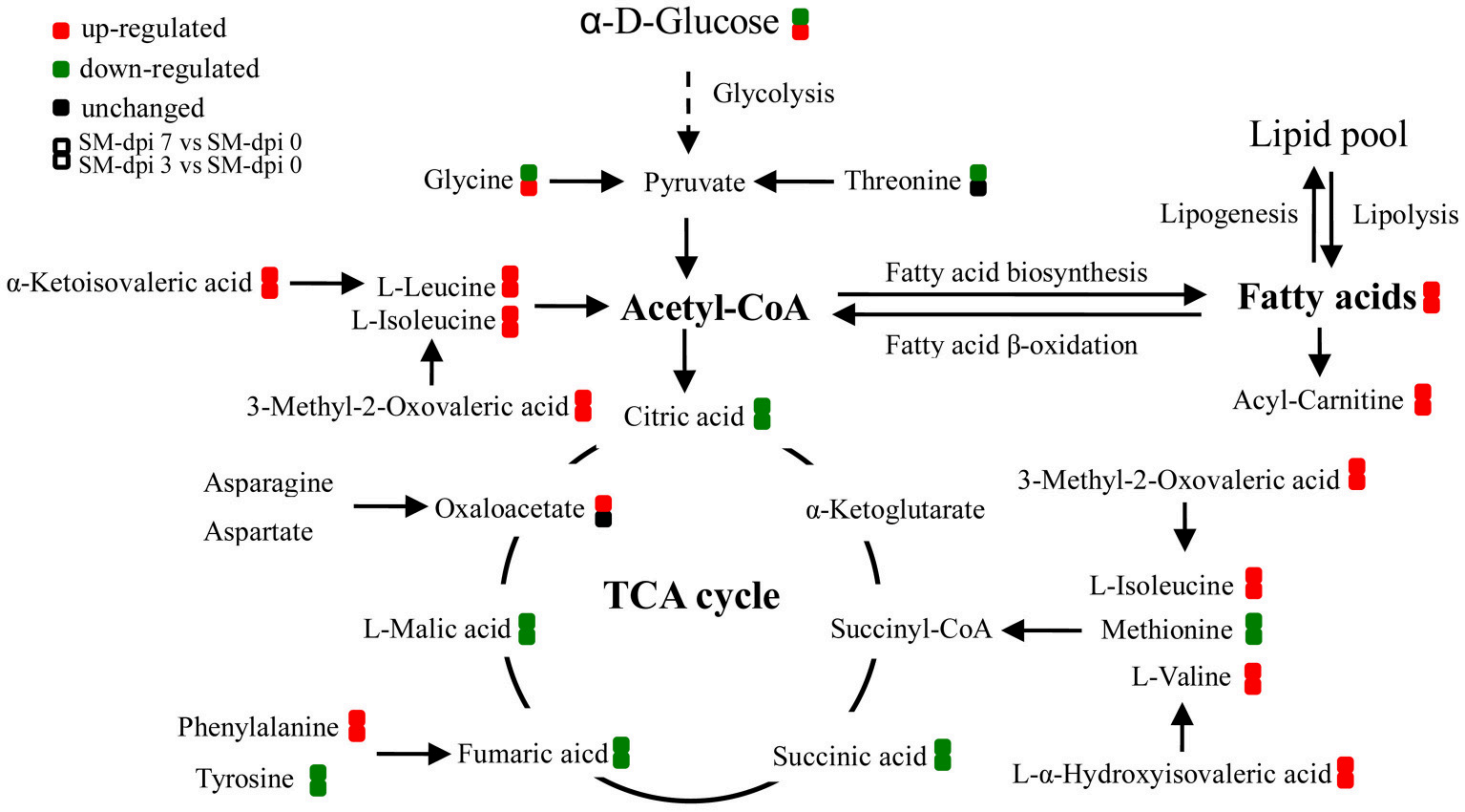
**Altered energy metabolism in piglets infected with CSFV**. During CSF infection (SM), metabolic pathways associated with energy metabolism including TCA cycle, fatty acid biosynthesis, and β-oxidation, branched amino acids were significantly altered. Red: up-regulation; green: down-regulation.

Several pathways involved in energy metabolism were found to be perturbed during CSFV infection, including the TCA cycle, fatty acid biosynthesis and β-oxidation, BCAA degradation, and the metabolism of other amino acids (Figure 5). The serum levels of citric, succinic, fumaric, and malic acids, which are TCA cycle intermediates for ATP production, were decreased during the infection period, indicating that the highly virulent CSFV Shimen strain can inhibit the energy-producing TCA cycle. Another energy pathway for acetyl-CoA production, fatty acid β-oxidation, was also suppressed immediately following CSFV infection, as evidenced by the substantial accumulation of long chain saturated and unsaturated fatty acids, carnitine, and acylcarnitines.

In addition, elevated levels of long chain fatty acids suggest that CSFV infection promotes fatty acid biosynthesis, which may in turn facilitate the formation of membranous viral replication complexes and promote viral propagation. It has been reported that dengue infection also causes the accumulation of long chain fatty acids and acylcarinitines in the serum of patients (Sun et al., 2013). Indeed, several viruses within the family *Flaviviradae* can hijack the fatty acid biosynthesis pathway and impair β-oxidation. Dengue virus (DENV) infection redistributes fatty acid synthase to viral replication sites through interaction between non-structural protein 3 (NS3) and fatty acid synthase in order to promote cellular fatty acid synthesis (Heaton et al., 2010). In addition, DENV replication can also be enhanced by Rab18 through targeting fatty acid synthase to sites of viral replication (Tang et al., 2014). In hepatitis C, hepatitis C virus (HCV) infection not only upregulates the expression of fatty acid synthase but also attenuates mitochondrial β-oxidation through down-regulation of mitochondrial trifunctional protein (MTP) (Yang et al., 2008; Amako et al., 2015). This situation is similar to Japanese encephalitis viral infection, which impairs fatty acid β-oxidation through interaction between viral non-structural protein 5 (NS5) and hydroxyacyl-CoA dehydrogenase α and β subunits, two components of MTP involved in long chain fatty acid β-oxidation (Kao et al., 2015). The underlying mechanisms of fatty acid biosynthesis and β-oxidation hijacked by CSFV infection therefore merit further investigation.

BCAAs and their derivative BCFAs are also important sources of energy production as are other amino acids including methionine and threonine (Guo et al., 2015; Neis et al., 2015). Degradation of these produces acetyl-CoA and/or TCA cycle intermediates for energy production. In general, BCAAs are broken down by the action of multifunctional branched chain α-ketoacid dehydrogenase complex (BCKDH), which converts them into acyl-CoA derivatives and subsequently into acetyl-CoA or succinyl-CoA that enter the TCA cycle (Shimomura et al., 2006). A deficiency of BCKDH causes the accumulation of BCAAs and their toxic by-product BCFAs (L-α-hydroxyisovaleric acid, α-ketoisovaleric acid, and 3-methyl-2-oxovaleric acid) in the blood and urine, resulting in a condition known as maple syrup urine disease (MSUD), which accumulates damage in mitochondrial DNA and nuclear DNA and subsequently impairs mitochondrial biogenesis (Aevarsson et al., 2000; Strand et al., 2014). Symptoms of MSUD, including anorexia, vomiting, dehydration, seizures, and coma have been commonly observed in infected piglets following CSFV infection, indicating the potential involvement of increased levels of BCAAs and their derivatives in the disease progression. Since BCAAs are essential amino acids and cannot be synthesized by host, but bacteria, which carry genes encoding multiple acetohydroxy acid synthase (AHAS) isozymes, can catalyze the production of acetolactate (a precursor of valine, leucine, and co-enzyme A) through decarboxylation of pyruvate and its condensation either with a second molecule of pyruvate, or with 2-ketobutyrate to produce acetohydroxybutyrate (a precursor of isoleucine) (Epelbaum et al., 1998), and degradation of BCAAs can also be performed by bacteria (Massey et al., 1976). Therefore, it appears that inhibition of BCAA degradation not only leads to suppression of energy production but also contributes to disease development in CSFV-infected piglets. However, it remains undetermined how CSFV infection regulates the expression or activity of BCKDH in piglet and AHAS in gut bacteria and then suppresses the degradation of BCAAs.

We found that aromatic amino acid metabolites, including serotonin, kynurenine, indole-containing metabolites, phenylalanine, and equol, were significantly perturbed in infected animals. Serotonin, kynurenine, and indole-containing metabolites are tryptophan derivatives, alteration of these metabolites indicates that CSFV infection changed tryptophan metabolism. Serotonin is a well-documented neurotransmitter and plays an important role in the brain-gut microbiome axis, which has been implicated in gastrointestinal pathologies such as irritable bowel syndrome and Crohn's disease (O'Mahony et al., 2015), which can cause depression, alternative constipation and diarrhea, the common clinical signs of CSF.

The tryptophan-kynurenine pathway catabolizes tryptophan to produce NAD. In eukaryotes catabolism of tryptophan to kynurenine is the rate-limiting step in this pathway. Intermediates in the kynurenine pathway such as kynurenine and quinolinic acid are known to induce the apoptosis of lymphocytes, especially Th1 and NK cells, inhibition of T-cell proliferation, enhancement of regulatory T-cell proliferation, and inhibition of the production of type I interferon (IFN-I) (Fallarino et al., 2002; Boasso et al., 2007; Hoshi et al., 2010, 2012; Song et al., 2011). High activity of indoleamine 2,3-dioxygenase (IDO) has been reported to be associated with renal insufficiency in nephropathia epidemica (Puumala hantavirus infection) (Outinen et al., 2011). It is well-known that leukopenia and inhibition of IFN-I production are typical features of CSFV infection, and since marked increases in kynurenine and quinolinic acid were observed at dpi 3 with a peak at dpi 7, the CSFV-activated kynurenine pathway may serve as a critical contributor to the disease progression of CSF. In addition to the eukaryotic tryptophan-to-quinolinate pathway, the biosynthesis of quinolinic acid can also be performed by the bacterial aspartate-to-quinolinate pathway, which was reported to be involved in the pathogenesis of HIV infection (Lima et al., 2009; Vujkovic-Cvijin et al., 2013). Thus, studies should be performed in future to test whether the elevated kynurenine and quinolinate levels in the sera of CSFV-infected piglets are associated with the gut microbiota and subsequently cause immunosuppression and renal failure.

Other indole-containing metabolites were also affected by CSFV infection, including elevation of indoxylsulfinic acid and significantly down-regulation of 3-IPA and indoleacetaldehyde. Indoxylsulfinic acid has been identified as being generated only by gut microbiota (Wikoff et al., 2009). It is a known uremic toxin that accumulates in the blood of patients suffering from chronic kidney disease and arises from hepatic transformation of the bacterial metabolite indole (Deguchi et al., 2002). The accumulation of indoxysulfinic acid together with elevated urea, *p*-cresol sulfate, uric acid, allantoin, creatine, and creatinine strongly associate with the occurrence of uremia in CSF (Deguchi et al., 2002; Meyer and Hostetter, 2012), which can cause signs and symptoms within multiple systems (Meyer and Hostetter, 2007), e.g., coma, superficial gastrointestinal ulcers and pulmonary edema, all of which are commonly observed during CSF. Thus, altered metabolites associated with uremia may contribute to the disease development of CSF and the occurrence of kidney failure. 3-IPA is a powerful antioxidant and metabolite of indole, the production of which is completely dependent on the presence of gut microbiota and can be established by colonization with the bacterium *Clostridium sporogenes* (Wikoff et al., 2009). In addition, metabolism of aromatic amino acids phenylalanine and its derivatives and the flavone metabolite, equol, which were altered in CSFV-infected piglets, was also reported to be regulated by bacteria (Parent et al., 2002; Wikoff et al., 2009; Lu et al., 2012; Meyer and Hostetter, 2012). Taken together, altered levels of aromatic amino acids and other differential metabolites in the serum of infected piglets may provide an indication of intestinal dysbiosis induced by CSFV infection, which will be confirmed in future studies.

In summary, we have analyzed the serum metabolomics of CSFV-infected piglets and shown that changes in the levels of certain metabolites can separate infected animals from healthy ones. Perturbed metabolic pathways include the tryptophan/kynurenine pathway, fatty acid biosynthesis and β-oxidation, the TCA, and urea cycles, and nucleotide metabolism. Impairment of these pathways has revealed that inhibition of energy production and possibly dysbiosis of the gut microbiota are critical contributors to the disease development of CSF. Our studies provide direct evidence that CSFV infection can cause significant alteration of lipid metabolism and many other metabolic pathways, similar to that occurs in human infections caused by DENV and HCV. Thus, metabolomics profiling of CSFV-infected piglets not only provides new insights into the disease progression of CSF, but also may serve as a model for evaluating therapeutics targeting flavivirus infection in humans.

## Author contributions

WG and JJ contributed to design of the study and draft the manuscript, BZ, SM, LZ, and XX contributed to sample collection and data analysis, HG, JS, and CT critically revised the manuscript.

## Funding

This work was supported by the following grants: National Natural Science Foundation of China to WG (No. 31572528) and to CT (No. 31130052), and China Postdoctoral Science Foundation project to WG (No. 2013M532129). WG and JS were also partially supported by a National Bio- and Argo-defense Facility Transition award.

### Conflict of interest statement

The authors declare that the research was conducted in the absence of any commercial or financial relationships that could be construed as a potential conflict of interest.
